# Do black lives matter in public health research and training?

**DOI:** 10.1371/journal.pone.0185957

**Published:** 2017-10-10

**Authors:** Molly Rosenberg, Shabbar I. Ranapurwala, Ashley Townes, Angela M. Bengtson

**Affiliations:** 1 Department of Epidemiology and Biostatistics, Indiana University School of Public Health-Bloomington, Bloomington, Indiana, United States of America; 2 Department of Epidemiology, Gillings School of Global Public Health, University of North Carolina-Chapel Hill, Chapel Hill, North Carolina, United States of America; 3 Department of Applied Health Sciences, Indiana University School of Public Health-Bloomington, Bloomington, Indiana, United States of America; The Chinese University of Hong Kong, HONG KONG

## Abstract

**Objective:**

To examine whether investments made in public health research align with the health burdens experienced by white and black Americans.

**Methods:**

In this cross-sectional study of all deaths in the United States in 2015, we compared the distribution of potential years of life lost (PYLL) across 39 causes of death by race and identified key differences. We examined the relationship between cause-of-death-specific PYLL and key indicators of public health investment (federal funding and number of publications) by race using linear spline models. We also compared the number of courses available at the top schools of public health relevant to the top causes of death contributor to PYLL for black and white Americans.

**Results:**

Homicide was the number one contributor to PYLL among black Americans, while ischemic heart disease was the number one contributor to PYLL among white Americans. Firearm-related violence accounted for 88% of black PYLL attributed to homicide and 71% of white PYLL attributed to homicide. Despite the high burden of PYLL, homicide research was the focus of few federal grants or publications. In comparison, ischemic heart disease garnered 341 grants and 594 publications. The number of public health courses available relevant to homicide (n = 9) was similar to those relevant to ischemic heart disease (n = 10).

**Conclusions:**

Black Americans are disproportionately affected by homicide, compared to white Americans. For both black and white Americans, the majority of PYLL due to homicide are firearm-related. Yet, homicide research is dramatically underrepresented in public health research investments in terms of grant funding and publications, despite available public health training opportunities. If left unchecked, the observed disproportionate distribution of investments in public health resources threatens to perpetuate a system that disadvantages black Americans.

## Introduction

The goal of public health is to protect and promote the health of all people.[1] The existence of persistent racial health disparities between black and white Americans is at stark odds with this goal. Racial health disparities occur when the distribution of health conditions and health outcomes is uneven by race, exemplified in the intractable life expectancy gap between black and white Americans.[2] The Healthy People 2010 and 2020 initiatives,[3] designed to eliminate health disparities in the US, have been largely ineffective in reducing racial disparities in many areas like physical activity, substance abuse, and healthcare access.(4) Racial disparities in areas like injury and violence have actually increased over time.[4] To reduce racial health disparities and to improve the misalignment between public health goals and the reality of the American public health landscape, it is critical to identify and address the underlying structural reasons that disadvantage black American’s health, as compared to white American’s health.[5]

A key structural indicator of public health priorities is the investments we make in public health research. Investments in public health research could contribute to racial health disparities if they systematically advantage or disadvantage certain racial groups. Public health research creates the evidence base for effective health policy[6] and for medical breakthroughs that can lead to rapid improvements in disease and injury treatment and prevention.[7–9] However, not all health conditions are studied equally and conditions that represent larger public health burdens tend to receive more attention. Further, external forces such as funding opportunities and training experiences could direct the focus of public health research towards or away from specific health conditions that may be disproportionately distributed across racial groups. Recently the Black Lives Matters movement has advocated against institutional racism that disadvantages black lives and health. Against that backdrop, several in the public health community have questioned if our research and research applications protect the health of all Americans equally.[10, 11]

In this analysis, we examine whether investments in public health research, in terms of federal grant funding and publications, accurately reflect the health issues of both black and white Americans. We quantify the relative importance of different health issues with potential years of life lost (PYLL), a measure that gives greater weight to the burden of premature deaths, and examine how PYLL for a range of causes of death (COD) relate to investments in public health research funding and publications.

## Methods

We calculated PYLL for each US death in 2015, reported by the Centers for Disease Control and Prevention (CDC).[12] Potential years of life lost (PYLL) measures the number of years a person would have lived if they had not died of a particular cause.[13] PYLL is a useful tool for comparing the burden of specific causes of death between groups, and highlights the burden premature deaths—which often have the greatest impact on a community. There are many measures that can be used to quantify the relative importance of different health outcomes (i.e. PYLL, crude mortality, and cause-specific morbidity), each with its own advantages and disadvantages. We chose to focus our analysis on PYLL because of its emphasis on premature death. In contrast, crude mortality rates are dominated by the health issues of the elderly without penalty for lengthy lifespan before death. Morbidity measures like prevalence and incidence do not take into account when death occurs in a person’s lifespan, making relative comparisons between conditions difficult. We posit that PYLL is reasonable measure for this paper’s focus because preventing premature death is a key public health goal with a direct impact of extending life expectancy and it should play a major role in setting research priorities.

PYLL was calculated for each death by subtracting the age at death from 75[13] and then summed by black and white race separately for each of the 39 CODs, defined by ICD-10 codes.[14] We present results for white and black racial categories inclusive of ethnic backgrounds, as results from analyses restricting to non-Hispanic white and black categories revealed similar findings. Eight ‘other’ causes of death were removed from some analyses to make comparisons across specific CODs. Homicide-attributed PYLL were further categorized as firearm-related or not. We compared the COD-specific PYLL for white and black Americans. To further understand the disparities in premature death among black and white Americans, we also calculated the average PYLL per death for each COD and the difference in average PYLL per death by race. The differences in PYLL per death between black and white Americans were ranked from highest to lowest.

To identify public health investments for each COD, we searched the Pubmed database to identify the number of publications associated with each specific COD. We excluded eight ‘other’ categories for a total of 31 causes of death searched. We used the Advanced search function to build our search strings, including the MeSH term(s) associated with each cause of death, a publication timeframe from January 1, 2015 to December 31, 2015, and a ‘United States’ MeSH term for each search so that the search results reflected publications relevant to US populations. The search string for homicide specifically excludes the MeSH term ‘Euthanasia.’ The MeSH terms we used for deaths due to ‘pregnancy, childbirth, and the puerperium’ were ‘maternal death’ OR ‘maternal mortality’. All search strings are recorded in S1 Table.

We also measured federal research funding investments by searching COD-specific keywords in National Institutes of Health (NIH) RePORTER[15] for R01 grants funded in 2015. We excluded eight ‘other’ categories for a total of 31 causes of death searched. NIH REPORTER is not cataloged according to MeSH terms, so we identified keywords to search adapted from the MeSH terms we used in the Pubmed search or from the major components of each category. We searched for all funded R01-level grants in the fiscal year 2015. We attempted to exclude research on non-US study populations by including ‘US,’ ‘United States,’ or ‘American’ search terms. All search fields are recorded in S1 Table.

We assessed the relationship between COD-specific PYLL with public health investments in federal research funding and publications by fitting linear regression models, coding PYLL using linear splines with knots at tertiles determined by the overall contribution to PYLL made by each COD. Graphical representations of these findings were stratified by race, plotting (i) PYLL per COD by the number of publications per COD and (ii) PYLL per COD by the number of R01s per COD. In these scatterplots, we compared the regression line predictions from the linear spline model to reference lines illustrating a 1% increase in PYLL corresponding to a 1% increase in publications and R01 grants.

To assess investments in the training of public health researchers, we also counted the number of public health courses that train new investigators to conduct research relevant to the top contributors to PYLL for white Americans and black Americans from the five most highly ranked American schools of public health.[16] Course catalogs from each school were searched for key words associated with the cause of death. Courses were counted as related to ischemic heart disease (top contributor to PYLL among white Americans) if they included ‘ischemic heart disease,’ ‘myocardial ischemia’, ‘coronary heart disease’, or ‘coronary artery disease’ in the course description. Courses were counted as related to gun violence/homicide (top contributor to PYLL among black Americans) if they included ‘homicide,’ ‘gun violence,’ or ‘firearm,’ anywhere in the course description. All calculations and analyses were conducted in SAS (v9.4). A copy of the SAS code used to produce the results of the analysis is included as a supplemental file (S1 File). All mortality data used were de-identified and publically available from the CDC. As such this analysis was deemed as non-human subjects research by the Indiana University Office of Research Compliance (#1705637870).

## Results

**Fig 1:**
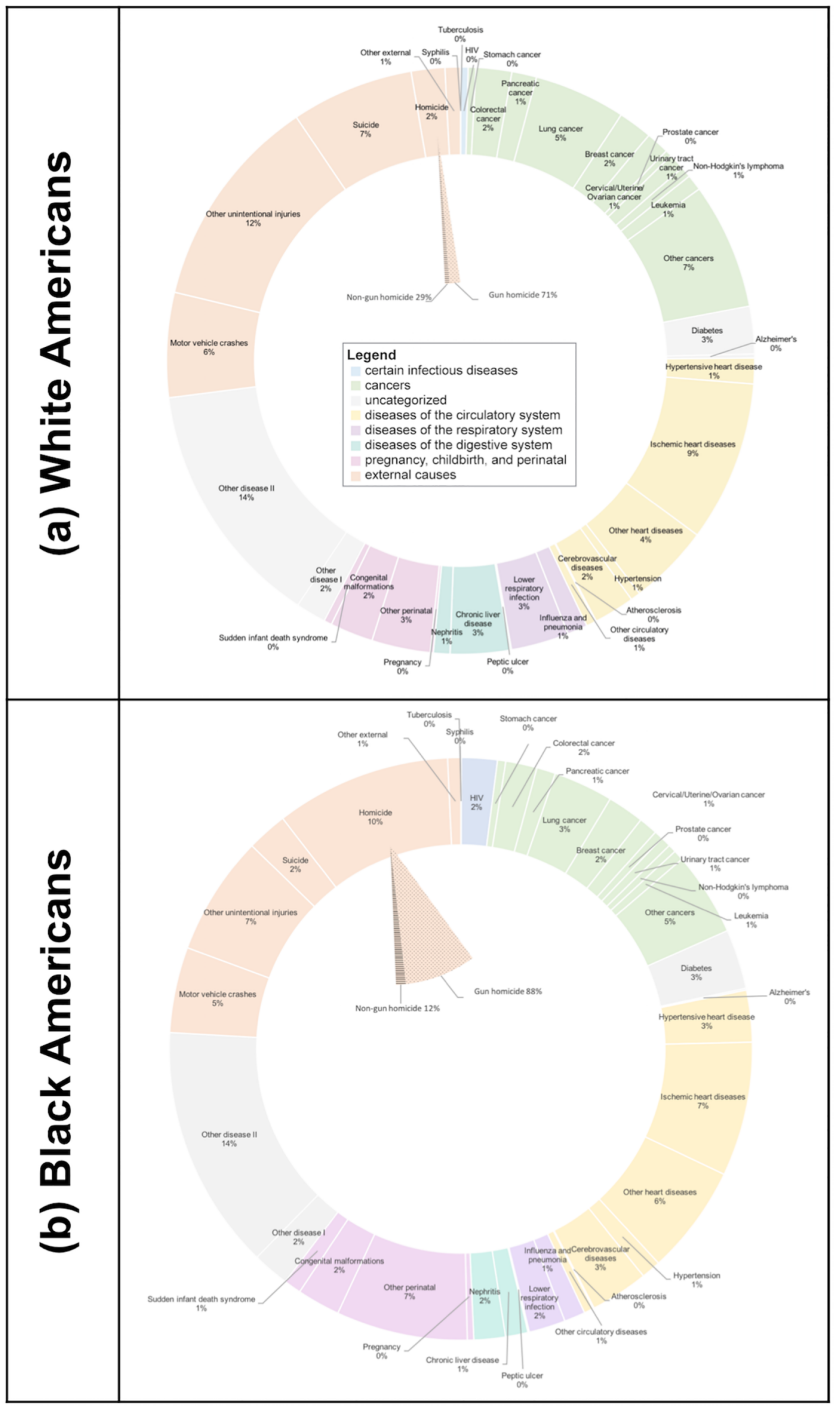
Proportion of potential years of life lost attributable to each of 39 causes of death, stratified by white and black race: United States 2015.

In 2015, there were 2,718,198 deaths in the US accounting for 21,396,995 total PYLL before the age of 75 years (Table 1). Over three-quarters (76%) of PYLL were among white Americans. Black Americans accounted for 20% of PYLL, despite representing only 13% of the 2015 population.[17] The top PYLL contributor was ischemic heart disease (9%) for white Americans and homicide (10%) for black Americans (Fig 1). Ischemic heart disease (IHD) was the second leading cause of death among black Americans and homicide was the 12^th^ leading cause of PYLL (out of 31) for white Americans. Firearm-related violence accounted for 88% of PYLL due to homicide in black Americans and 71% of PYLL due to homicide in white Americans.

**Table 1:** Number of deaths and potential years of life lost attributed to each of 39 causes of death in all Americans in 2015. Number of National Institute of Health R01 grants and scientific articles published on each specific cause of death (n = 31) in 2015 are also noted.

	Black deaths			White deaths			PYLL per death disparity (Black -White)	NIH R01s	Articles
Cause of death	N	PYLL	PYLL per death	N	PYLL	PYLL per death			
Tuberculosis	76	1065	14.0	275	2602	9.5	4.6	70	117
Syphilis	19	202	10.6	25	239	9.6	1.1	15	32
HIV	3458	83177	24.1	2905	65165	22.4	1.6	726	863
Stomach cancer	2028	19123	9.4	8358	68727	8.2	1.2	20	61
Colorectal cancers	7215	72743	10.1	43940	321608	7.3	2.8	170	334
Pancreatic cancer	5089	43854	8.6	35030	223014	6.4	2.3	83	80
Lung cancer	16313	138262	8.5	132925	821268	6.2	2.3	206	306
Breast cancer	6411	84713	13.2	34153	304575	8.9	4.3	359	559
Cervical/ Uterine/ Ovarian cancers	4079	43490	10.7	23019	207692	9.0	1.6	115	277
Prostate cancer	4750	20232	4.3	23393	59555	2.5	1.7	178	245
Urinary tract cancers	2697	23702	8.8	28194	144933	5.1	3.6	42	123
Non-Hodgkin’s lymphoma	1543	18702	12.1	17885	95934	5.4	6.8	36	59
Leukemia	1901	23865	12.6	20117	145183	7.2	5.3	216	99
Other cancers	17494	194372	11.1	139161	1134886	8.2	3.0		
Diabetes mellitus	13876	137978	9.9	62001	425013	6.9	3.1	926	857
Alzheimer’s disease	8157	4278	0.5	99889	35730	0.4	0.2	367	75
Hypertensive heart disease	9948	119988	12.1	35418	230498	6.5	5.6	19	35
Ischemic heart diseases	39840	311211	7.8	316579	1444520	4.6	3.2	341	594
Other heart diseases	25640	253588	9.9	189955	727868	3.8	6.1		
Hypertension	5820	45295	7.8	25047	89327	3.6	4.2	428	319
Cerebrovascular diseases	18027	134385	7.5	116969	365897	3.1	4.3	31	491
Atherosclerosis	540	2660	4.9	5402	9980	1.8	3.1	318	182
Other circulatory diseases	2354	21585	9.2	16669	82712	5.0	4.2		
Influenza and pneumonia	5651	48777	8.6	48960	180688	3.7	4.9	201	296
Chronic lower respiratory diseases	10488	84921	8.1	141882	519146	3.7	4.4	213	259
Peptic Ulcer	284	3266	11.5	2645	15585	5.9	5.6	6	2
Chronic liver diseases	3261	52150	16.0	35478	530714	15.0	1.0	74	149
Nephritis	9174	73131	8.0	39124	149467	3.8	4.2	30	23
Pregnancy	374	15081	40.3	701	27636	39.4	0.9	7	14
Other perinatal disease	4065	304635	74.9	7030	526047	74.8	0.1		
Congenital and chromosomal abnormalities	1661	99864	60.1	7890	386596	49.0	11.1	103	507
Sudden infant death syndrome	537	40275	75.0	948	71100	75.0	0.0	22	14
Other diseases I^a^	3843	85336	22.2	26233	270719	10.3	11.9		
Other diseases II^b^	55298	567235	10.3	441789	2227136	5.0	5.2		
Motor vehicle crashes	5485	198333	36.2	30844	939905	30.5	5.7	9	6
Other unintentional causes	10774	277755	25.8	97901	1929763	19.7	6.1		
Suicide	2509	94136	37.5	39908	1098977	27.5	10.0	93	301
Homicide	9193	407330	44.3	8072	300906	37.3	7.0	16	57
Other external causes	887	30197	34.0	4385	139463	31.8	2.2		
TOTAL	320759	4180892	13.0	2311099	16320774	7.1	6.0	5440	7336

^a^–includes symptoms, signs, and abnormal clinical and laboratory findings, not elsewhere classified.

^b^–includes all other residual causes of death.

**Fig 2:**
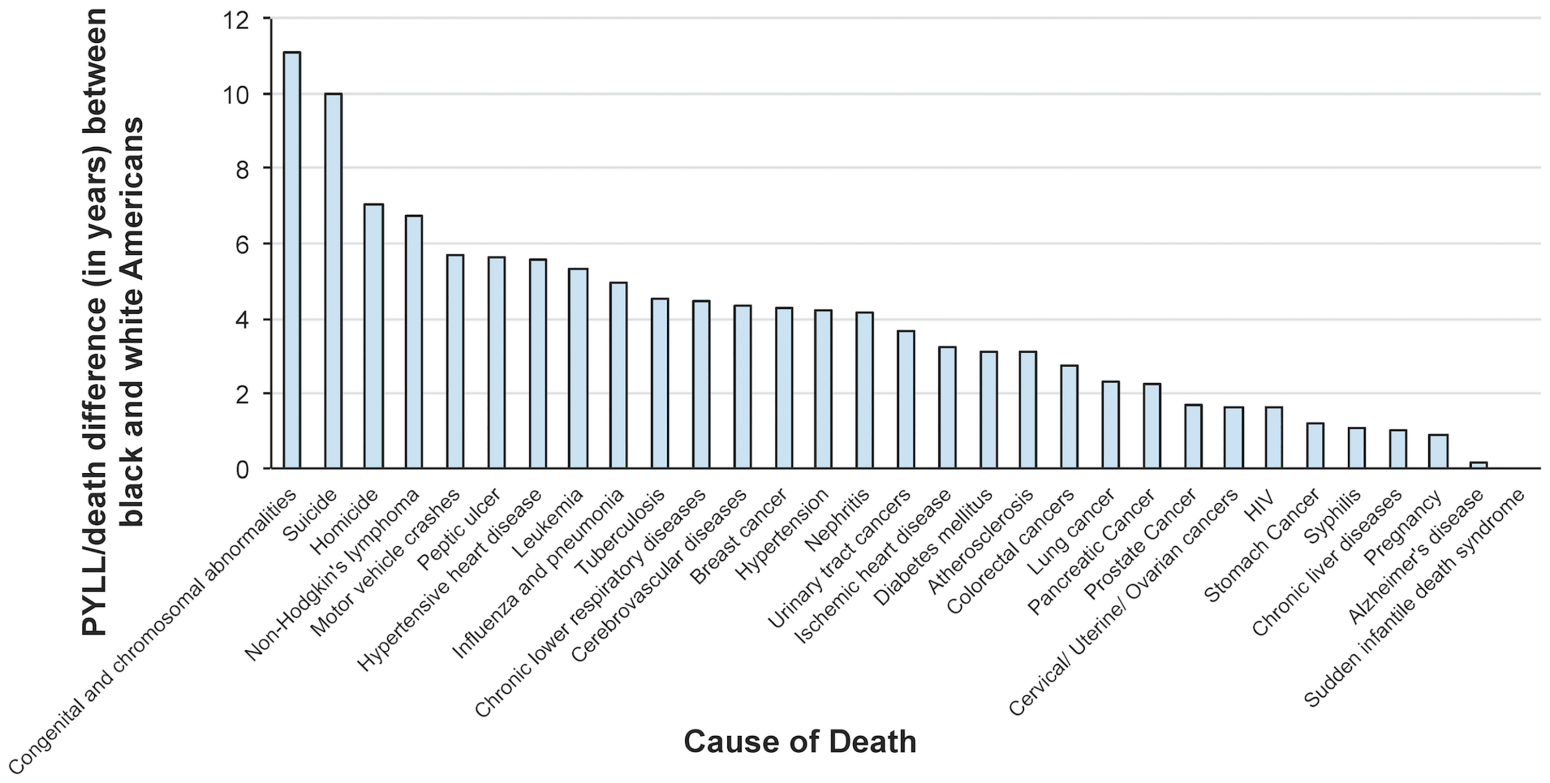
Disparity in PYLL per death attributed to each of 31 specific causes of death between black and white Americans in 2015.

Disparities existed in the number of PYLL per death between black Americans and white Americans for all CODs, cause-specific (n = 30) and non-specific (n = 8), except sudden infantile death syndrome (SIDS). SIDS only occurs in infants (age = 0 years), thus PYLL/death = 75 for both black and white infants (Table 1). The CODs that resulted in the largest disparities in average age at death between black and white Americans were congenital and chromosomal abnormalities, followed by suicide and homicide (Fig 2), both of which resulted in a greater absolute number of deaths and PYLL than congenital and chromosomal abnormalities (Table 1). The average black American dying from a congenital abnormality died at 15 years of age, compared to the average white American who died at age 26 from a similar condition. The average black American committing suicide was 10 years younger than the average white American (38 years old versus 48 years old). Homicide, the leading contributor to PYLL among black Americans, had the third highest racial disparity in average age at death (30 years old in black Americans versus 37 years old in white Americans). IHD, the leading contributor to PYLL among white Americans, ranked 17^th^ in terms of racial disparity in average age at death. Black Americans died on average 3 years earlier from IHD (average age at death 67 years), compared to white Americans (average age at death 70 years).

**Fig 3:**
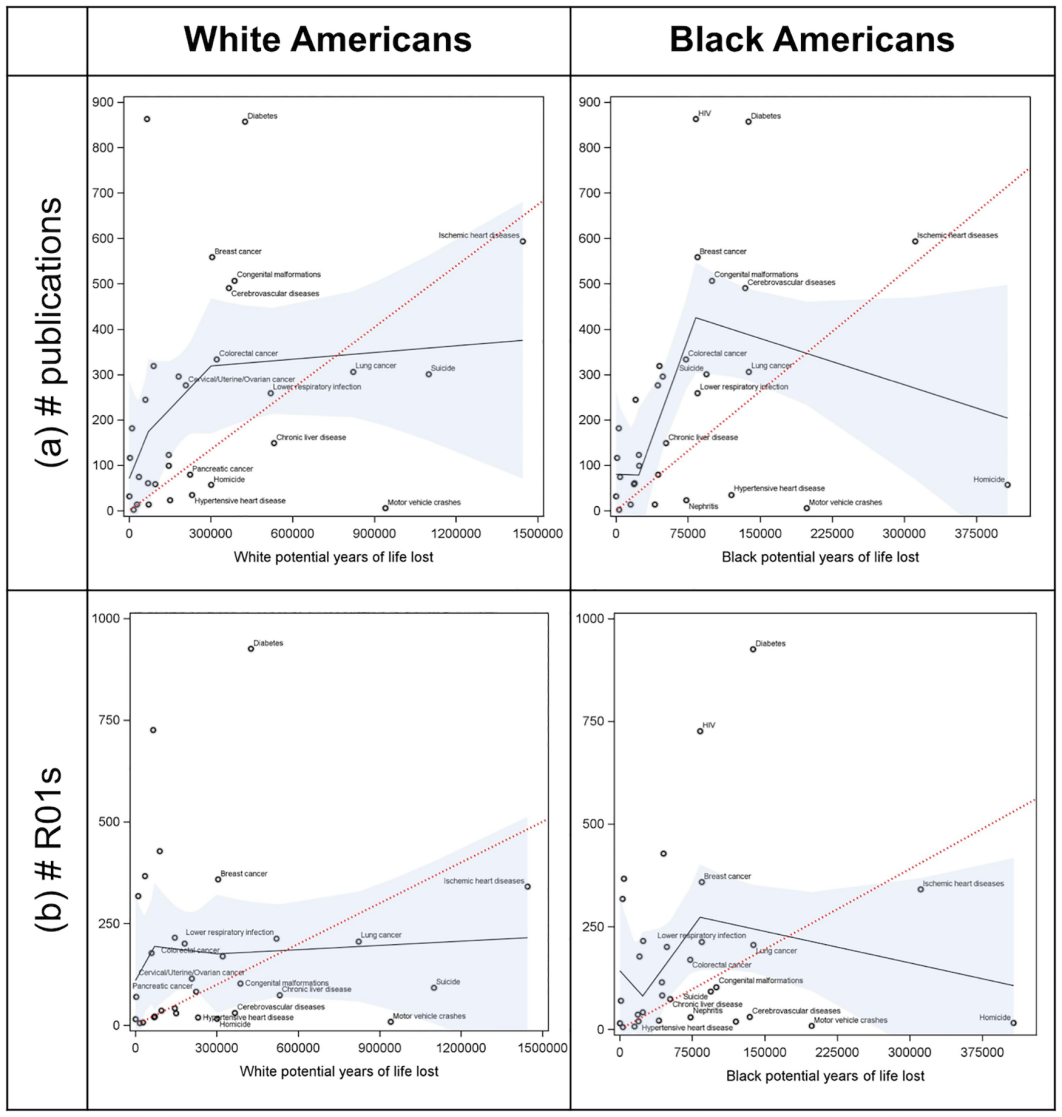
The relationship between the number of potential years of life lost (PYLL) due to 31 causes of death in all 2015 US deaths and (a) the number of journal articles written about the cause of death and (b) the number of NIH funded R01 grants about the cause of death, stratified by black and white race. Solid black line shows predicted relationships from linear spline regression models with nodes at tertiles and 95% confidence limits in blue. Dotted red reference line shows hypothetical relationship if every 1% increase in PYLL corresponded with a 1% increase in (a) publications and (b) funded grants. Top 15 race-specific contributors to PYLL are labeled in each panel.

The relationship between COD-specific PYLL and number of publications differed by race (Fig 3). Across the highest ranked CODs, the number of publications increased by 5 per 100,000 PYLL for white Americans, but decreased by 68 per 100,000 PYLL for black Americans. This difference is driven largely by homicide, the highest contributor to black PYLL but with few publications (n = 57). The highest contributor to white PYLL, IHD, garnered a large number (n = 594) of publications.

A similar trend was observed between COD-specific PYLL and number of NIH R01 grants. This disparity was again driven by homicides receiving few grants (n = 16) compared to IHD (n = 341 grants). The relationships between PYLL and publications/grants were not sensitive to the introduction of a one-year lag between deaths in 2014 and publications/funded grants in 2015 (S1 Fig), nor were they sensitive to restriction of the population to non-Hispanic ethnicity.

While there were large disparities in the funded research and publications pertaining to homicide and IHD, there were fewer apparent differences in the number of training opportunities relevant to these top contributors of PYLL for black and white Americans, respectively. Across the 41 departments in the top five schools of public health, nine courses mentioned homicide, gun violence, or firearms in the course description. Ten courses mentioned ischemic heart disease in the course description (Table 2).

**Table 2:** Number of courses offered at the top five American schools of public health with mention of firearm violence/homicide in course description.

School	Number of departments	Number of courses related to firearm violence/homicide	Number of courses related to ischemic heart disease
Johns Hopkins Bloomberg School of Public Health	10	2	3
Harvard T.H. Chan School of Public Health	9	1	2
University of North Carolina-Chapel Hill Gillings School of Global Public Health	8	3	4
University of Michigan School of Public Health	7	2	1
Columbia University Mailman School of Public Health	7	1	0
**TOTAL**	**41**	**9**	**10**

## Discussion

In this study, we found that homicide was the largest contributor to black PYLL and the 12^th^ leading contributor to white PYLL. The majority of homicides for both black and white Americans were firearm-related. Racial disparities in average age at death were observed across all 39 COD assessed but were among the largest for homicide-related deaths. Despite the high PYLL burden of homicide overall and the racial disparity observed, the number of publications and federally funded grants for homicide research were among the lowest we assessed.

Our findings align with long-standing disparities in life expectancy between black and white Americans.[2, 18] As far back as the 1980s, homicide has been noted as a major source of the racial disparities in life expectancy.[18] Like homicide, suicide may also be firearm-related. While suicides rates are lower among racial minorities, compared to white Americans,[19, 20] our findings highlight that suicide is also an important cause of premature death among black Americans. The gap in life expectancy between white and black Americans is shrinking; however, it remains at 5.4 years for men and 3.8 years for women.[2] While there are disparities between black and white Americans across all CODs, our results make clear that public health efforts to reduce US life expectancy disparities must include homicide and firearm research and prevention. This is supported by a recent study that suggests lack of funding for gun violence research relative to other causes of death.[21]

Lack of homicide research reflects the scarce federal funding for firearm violence research.[21] In 1996, in response to a CDC-funded study demonstrating that having a gun in the home increased the risk of homicide, pro-gun advocates successfully lobbied congress to reduce the CDC budget by $2.6 million—the exact amount devoted for firearm research the previous year.[22]. In 2012, this funding caveat was extended to the NIH, which continues to fund a small portfolio of firearm violence research. Congress further mandated that federal funds to the CDC not “be used to advocate or promote gun control”.[23] Despite calls for more firearm violence research funding, no specific funds have been appropriated by Congress to the CDC.[22] The lack of funding is likely a major factor in why homicide and firearm violence have not been seen as major public health problems.

However, we did not find evidence to suggest homicide research is poorly represented in public health coursework compared to ischemic heart disease, the top contributor to PYLL in white Americans. Although courses were available relevant to homicide and firearm violence, these were primarily injury epidemiology courses with broad course descriptions that included homicide or firearm violence but were not exclusively limited to the subject. A larger training issue is that public health students, even those trained in injury epidemiology, likely get very little hands-on experience with homicide or firearm research in mentored training environments given the limited amount of publications and R01 grants for homicide research we observed. That said, across the US, there are a handful of leading programs that conduct firearm-related violence research, including the Violence Prevention Research Program at University of California, Davis, the Johns’ Hopkins Center for Gun Policy and Research, and the Harvard Injury Control Research Center, in addition to initiatives like ‘Cure Violence’ (University of Illinois, Chicago). These programs and initiatives are primarily funded by foundations.

We chose PYLL as our key measure to identify the relative importance of causes of death; it has both advantages and disadvantages relative to other measures. In terms of disadvantages, PYLL does not capture morbidity, non-fatal injuries, or the value of lives lost after age 75. Other measures such as disease incidence, prevalence, or cause-specific crude mortality rates may well produce different patterns of results than we observed and should be evaluated in future research. Indeed, in examining the cause-specific crude mortality rates in our study, we find they differ from the top contributors to PYLL, and that the racial disparities are less apparent. However, a key strength of PYLL compared to other measures is its focus on premature death. Premature death, among its many negative consequences, exacts a toll of unreached potential societal productivity. The more premature a death, the greater the loss in economic productivity for the family, community, and society left behind.[24] For example, the estimated value of remaining lifetime productivity for a 31 year old American (the average age at homicide death for black Americans) is $1,521,239. In comparison, the estimated value of remaining lifetime productivity for a 70 year old American (the average age at ischemic heart disease death for white Americans) is $140,804.[24] Thus, in highlighting premature deaths, PYLL calls attention to deaths with high societal and economic impact.

To capture key population-level trends, our analysis was conducted with some broad categorizations that merit more granular study in future research. First, the categorization of all black Americans into one category does not recognize the diversity within this population nor the differences that may exist between other races. Second, even though we examined the number of IHD and homicide-related courses offered in the top schools of public health, we did not measure the number of enrolled students or available faculty in these areas, which may also be relevant measures of subject matter emphasis. Finally, important differences in homicide PYLL may also exist by gender or socioeconomic status and race, unexamined here.

Homicide-related deaths in America constitute a public health crisis. Black Americans are disproportionately affected by homicide and the majority of homicide PYLL for both black and white Americans is attributed to firearm-related violence. In addition to lives lost, large PYLL captures the loss of human potential that can push families into poverty and societies toward heightened inequality. There is a pressing need to increase funding for homicide and firearm research and to increase use of publicly available data to understand firearm-related homicide and its downstream consequences. Along with more funding, we recommend expanded training opportunities in homicide and firearm research to prepare the next generation of public health professionals to address this urgent public health need. Unless addressed, the lack of funding and training for homicide research will perpetuate a system that specifically disadvantages the health of black Americans.

## Supporting information

S1 Table Search strings used to identify number of publications and number of R01 grants for each of 31 specific causes of death. (DOCX)

S1 File SAS code used to produce the results of study analysis. (SAS)

S1 Fig The one-year lagged relationship between the number of potential years of life lost (PYLL) due to 31 causes of US deaths in 2014 and (a) the number of journal articles written about the cause of death in 2015 and (b) the number of NIH funded R01 grants about the cause of death in 2015, stratified by black and white race. Solid black line shows predicted relationships from linear spline regression models with nodes at tertiles and 95% confidence limits in blue. Dotted red reference line shows hypothetical relationship if every 1% increase in PYLL corresponded with a 1% increase in (a) publications and (b) funded grants. Top 15 race-specific contributors to PYLL are labeled in each panel.
(TIFF)
