# Supplementary material for: Do black lives matter in public health research and training?
Source: PLoS One. 2017 Oct 10;12(10):e0185957. doi: 10.1371/journal.pone.0185957 (PMC5634659; doi:10.1371/journal.pone.0185957)
Supplement: S1 Table — (DOCX) [file pone.0185957.s001.docx]

**Supplemental Table 1.** Search strings used to identify number of publications and number of R01 grants for each of 31 specific causes of death

| **Cause of death** | **PubMed Advanced Search string** | **NIH Reporter Search terms** |
| --- | --- | --- |
| Tuberculosis | (("2015/01/01"[Date - Publication] : "2015/12/31"[Date - Publication])) AND "tuberculosis"[MeSH Major Topic] AND "United States"[MeSH Terms] | Text Search: Tuberculosis AND ("United States" OR "US" OR "American" OR "USA") (Advanced), Search in: Projects Admin IC: All, Activity Code: R01, Fiscal Year: 2015 |
| Syphilis | (("2015/01/01"[Date - Publication] : "2015/12/31"[Date - Publication])) AND "syphilis"[MeSH Major Topic] AND "United States"[MeSH Terms] | Text Search: Syphilis AND ("United States" OR "US" OR "American" OR "USA") (Advanced), Search in: Projects Admin IC: All, Activity Code: R01, Fiscal Year: 2015 |
| HIV | (("2015/01/01"[Date - Publication] : "2015/12/31"[Date - Publication])) AND ("hiv infections"[MeSH Major Topic] OR "hiv"[MeSH Major Topic] ) AND "United States"[MeSH Terms] | Text Search: HIV AND ("United States" OR "US" OR "American" OR "USA") (Advanced), Search in: Projects Admin IC: All, Activity Code: R01, Fiscal Year: 2015 |
| Malignant neoplasm of stomach | (("2015/01/01"[Date - Publication] : "2015/12/31"[Date - Publication])) AND "stomach neoplasms"[MeSH Major Topic] AND "United States"[MeSH Terms] | Text Search: "Stomach cancer" AND ("United States" OR "US" OR "American" OR "USA") (Advanced), Search in: Projects Admin IC: All, Activity Code: R01, Fiscal Year: 2015 |
| Malignant neoplasm of colon, rectum, anus | (("2015/01/01"[Date - Publication] : "2015/12/31"[Date - Publication])) AND ("Anus Neoplasms"[MeSH Major Topic] OR "Colorectal Neoplasms"[MeSH Major Topic] ) AND "United States"[MeSH Terms] | Text Search: ("Colon Cancer" OR "Rectal Cancer" OR "Colorectal Cancer") AND ("United States" OR "US" OR "American" OR "USA") (Advanced), Search in: Projects Admin IC: All, Activity Code: R01, Fiscal Year: 2015 |
| Malignant neoplasm of pancreas | (("2015/01/01"[Date - Publication] : "2015/12/31"[Date - Publication])) AND "pancreatic neoplasms"[MeSH Major Topic] AND "United States"[MeSH Terms] | Text Search: "Pancreatic cancer" AND ("United States" OR "US" OR "American" OR "USA") (Advanced), Search in: Projects Admin IC: All, Activity Code: R01, Fiscal Year: 2015 |
| Malignant neoplasm of trachea, bronchus, lung | (("2015/01/01"[Date - Publication] : "2015/12/31"[Date - Publication])) AND "Respiratory Tract Neoplasms"[MeSH Major Topic] AND "United States"[MeSH Terms] | Text Search: ("Tracheal Cancer" OR "Bronchial Cancer" OR "Lung Cancer") AND ("United States" OR "US" OR "American" OR "USA") (Advanced), Search in: Projects Admin IC: All, Activity Code: R01, Fiscal Year: 2015 |
| Malignant neoplasm of breast | (("2015/01/01"[Date - Publication] : "2015/12/31"[Date - Publication])) AND "Breast Neoplasms"[MeSH Major Topic] AND "United States"[MeSH Terms] | Text Search: "Breast cancer" AND ("United States" OR "US" OR "American" OR "USA") (Advanced), Search in: Projects Admin IC: All, Activity Code: R01, Fiscal Year: 2015 |
| Malignant neoplasm of cervix uteri, corpus uteri, ovary | (("2015/01/01"[Date - Publication] : "2015/12/31"[Date - Publication])) AND ("Uterine Cervical Neoplasms"[MeSH Major Topic] OR "Uterine Neoplasms"[MeSH Major Topic]OR "Ovarian Neoplasms"[MeSH Major Topic] ) AND "United States"[MeSH Terms] | Text Search: ("Uterine Cancer" OR "Cervical Cancer" OR "Ovarian Cancer") AND ("United States" OR "US" OR "American" OR "USA") (Advanced), Search in: Projects Admin IC: All, Activity Code: R01, Fiscal Year: 2015 |
| Malignant neoplasm of prostate | (("2015/01/01"[Date - Publication] : "2015/12/31"[Date - Publication])) AND "Prostatic Neoplasms"[MeSH Major Topic] AND "United States"[MeSH Terms] | Text Search: "Prostate Cancer" AND ("United States" OR "US" OR "American" OR "USA") (Advanced), Search in: Projects Admin IC: All, Activity Code: R01, Fiscal Year: 2015 |
| Malignant neoplasm of urinary tract | (("2015/01/01"[Date - Publication] : "2015/12/31"[Date - Publication])) AND "Urologic Neoplasms"[MeSH Major Topic] AND "United States"[MeSH Terms] | Text Search: ("Bladder Cancer" OR "Kidney Cancer" OR "Urethral Cancer") AND ("United States" OR "US" OR "American" OR "USA") (Advanced), Search in: Projects Admin IC: All, Activity Code: R01, Fiscal Year: 2015 |
| Non-Hodgkin's lymphoma | (("2015/01/01"[Date - Publication] : "2015/12/31"[Date - Publication])) AND "Lymphoma, Non-Hodgkin"[MeSH Major Topic] AND "United States"[MeSH Terms] | Text Search: "Non-Hodgkin's Lymphoma" AND ("United States" OR "US" OR "American" OR "USA") (Advanced), Search in: Projects Admin IC: All, Activity Code: R01, Fiscal Year: 2015 |
| Leukemia | (("2015/01/01"[Date - Publication] : "2015/12/31"[Date - Publication])) AND "Leukemia"[MeSH Major Topic] AND "United States"[MeSH Terms] | Text Search: (leukemia NOT hiv) AND ("United States" OR "US" OR "American" OR "USA") (Advanced), Search in: Projects Admin IC: All, Activity Code: R01, Fiscal Year: 2015^1^ |
| Diabetes mellitus | (("2015/01/01"[Date - Publication] : "2015/12/31"[Date - Publication])) AND "Diabetes Mellitus"[MeSH Major Topic] AND "United States"[MeSH Terms] | Text Search: diabetes AND ("United States" OR "US" OR "American" OR "USA") (Advanced), Search in: Projects Admin IC: All, Activity Code: R01, Fiscal Year: 2015 |
| Alzheimer's disease | (("2015/01/01"[Date - Publication] : "2015/12/31"[Date - Publication])) AND "Alzheimer disease"[MeSH Major Topic] AND "United States"[MeSH Terms] | Text Search: Alzheimer AND ("United States" OR "US" OR "American" OR "USA") (Advanced), Search in: Projects Admin IC: All, Activity Code: R01, Fiscal Year: 2015 |
| Hypertensive heart disease with or without renal disease | (("2015/01/01"[Date - Publication] : "2015/12/31"[Date - Publication])) AND ("Hypertension"[MeSH Major Topic] AND "Heart diseases"[MeSH Major Topic] ) AND "United States"[MeSH Terms] | Text Search: "Hypertensive heart disease" AND ("United States" OR "US" OR "American" OR "USA") (Advanced), Search in: Projects Admin IC: All, Activity Code: R01, Fiscal Year: 2015 |
| Ischemic heart diseases | (("2015/01/01"[Date - Publication] : "2015/12/31"[Date - Publication])) AND "Myocardial Ischemia"[MeSH Major Topic] AND "United States"[MeSH Terms] | Text Search: ("Ischemic heart disease" OR "Myocardial ischemia" OR "coronary heart disease" OR "coronary artery disease") AND ("United States" OR "US" OR "American" OR "USA") (Advanced), Search in: Projects Admin IC: All, Activity Code: R01, Fiscal Year: 2015 |
| Essential (primary) hypertension and hypertensive renal disease | (("2015/01/01"[Date - Publication] : "2015/12/31"[Date - Publication])) AND "Hypertension"[MeSH Major Topic] AND "United States"[MeSH Terms] | Text Search: Hypertension AND ("United States" OR "US" OR "American" OR "USA") (Advanced), Search in: Projects Admin IC: All, Activity Code: R01, Fiscal Year: 2015 |
| Cerebrovascular diseases | (("2015/01/01"[Date - Publication] : "2015/12/31"[Date - Publication])) AND "Cerebrovascular disorders"[MeSH Major Topic] AND "United States"[MeSH Terms] | Text Search: "Cerebrovascular disorders" AND ("United States" OR "US" OR "American" OR "USA") (Advanced), Search in: Projects Admin IC: All, Activity Code: R01, Fiscal Year: 2015 |
| Atherosclerosis | (("2015/01/01"[Date - Publication] : "2015/12/31"[Date - Publication])) AND "Atherosclerosis"[MeSH Major Topic] AND "United States"[MeSH Terms] | Text Search: Atherosclerosis AND ("United States" OR "US" OR "American" OR "USA") (Advanced), Search in: Projects Admin IC: All, Activity Code: R01, Fiscal Year: 2015 |
| Influenza and pneumonia | (("2015/01/01"[Date - Publication] : "2015/12/31"[Date - Publication])) AND ("Influenza, Human"[MeSH Major Topic] OR "Pneumonia"[MeSH Major Topic] ) AND "United States"[MeSH Terms] | Text Search: (Pneumonia OR Influenza) AND ("United States" OR "US" OR "American" OR "USA") (Advanced), Search in: Projects Admin IC: All, Activity Code: R01, Fiscal Year: 2015 |
| Chronic lower respiratory diseases | (("2015/01/01"[Date - Publication] : "2015/12/31"[Date - Publication])) AND ("Bronchitis"[MeSH Major Topic] OR "Bronchiectasis"[MeSH Major Topic] OR "Asthma"[MeSH Major Topic] OR "Emphysema"[MeSH Major Topic]) AND "United States"[MeSH Terms] | Text Search: (bronchitis OR bronchiectasis OR asthma OR emphysema) AND ("United States" OR "US" OR "American" OR "USA") (Advanced), Search in: Projects Admin IC: All, Activity Code: R01, Fiscal Year: 2015 |
| Peptic Ulcer | (("2015/01/01"[Date - Publication] : "2015/12/31"[Date - Publication])) AND "Peptic Ulcer"[MeSH Major Topic] AND "United States"[MeSH Terms] | Text Search: "peptic ulcer" AND ("United States" OR "US" OR "American" OR "USA") (Advanced), Search in: Projects Admin IC: All, Activity Code: R01, Fiscal Year: 2015 |
| Chronic liver disease and cirrhosis | (("2015/01/01"[Date - Publication] : "2015/12/31"[Date - Publication])) AND ("Liver Diseases, Alcoholic"[MeSH Major Topic] OR "Hepatitis, Chronic"[MeSH Major Topic] OR "Liver Cirrhosis"[MeSH Major Topic]) AND "United States"[MeSH Terms] | Text Search: ("alcoholic liver disease" OR "chronic hepatitis" OR "liver cirrhosis") AND ("United States" OR "US" OR "American" OR "USA") (Advanced), Search in: Projects Admin IC: All, Activity Code: R01, Fiscal Year: 2015 |
| Nephritis, nephrotic syndrome, and nephrosis | (("2015/01/01"[Date - Publication] : "2015/12/31"[Date - Publication])) AND ("Nephritis"[MeSH Major Topic] OR "Nephrotic syndrome"[MeSH Major Topic] OR "Nephrosis"[MeSH Major Topic]) AND "United States"[MeSH Terms] | Text Search: (nephritis OR "nephrotic syndrome" OR nephrosis) AND ("United States" OR "US" OR "American" OR "USA") (Advanced), Search in: Projects Admin IC: All, Activity Code: R01, Fiscal Year: 2015 |
| Pregnancy, childbirth, and the puerperium | (("2015/01/01"[Date - Publication] : "2015/12/31"[Date - Publication])) AND ("Maternal death"[MeSH Major Topic] OR "Maternal mortality"[MeSH Major Topic]) AND "United States"[MeSH Terms] | Text Search: ("maternal mortality" OR "maternal death") AND ("United States" OR "US" OR "American" OR "USA") (Advanced), Search in: Projects Admin IC: All, Activity Code: R01, Fiscal Year: 2015 |
| Congenital malformations, deformations, and chromosomal abnormalities | (("2015/01/01"[Date - Publication] : "2015/12/31"[Date - Publication])) AND ("Congenital abnormalities"[MeSH Major Topic]) AND "United States"[MeSH Terms] | Text Search: ("congenital abnormalities" OR "congenital abnormality") AND ("United States" OR "US" OR "American" OR "USA") (Advanced), Search in: Projects Admin IC: All, Activity Code: R01, Fiscal Year: 2015 |
| Sudden infant death syndrome | (("2015/01/01"[Date - Publication] : "2015/12/31"[Date - Publication])) AND "Sudden Infant Death"[MeSH Major Topic] AND "United States"[MeSH Terms] | Text Search: "sudden infant death" AND ("United States" OR "US" OR "American" OR "USA") (Advanced), Search in: Projects Admin IC: All, Activity Code: R01, Fiscal Year: 2015 |
| Motor vehicle crashes | (("2015/01/01"[Date - Publication] : "2015/12/31"[Date - Publication])) AND "motor vehicle accident" AND "United States"[MeSH Terms] | Text Search: "motor vehicle accident" AND ("United States" OR "US" OR "American" OR "USA") (Advanced), Search in: Projects Admin IC: All, Activity Code: R01, Fiscal Year: 2015 |
| Suicide | (("2015/01/01"[Date - Publication] : "2015/12/31"[Date - Publication])) AND "suicide"[MeSH Major Topic] AND "United States"[MeSH Terms] | Text Search: suicide AND ("United States" OR "US" OR "American" OR "USA") (Advanced), Search in: Projects Admin IC: All, Activity Code: R01, Fiscal Year: 2015 |
| Assault (homicide) | (("2015/01/01"[Date - Publication] : "2015/12/31"[Date - Publication])) AND ("homicide"[MeSH Major Topic] NOT "euthanasia"[MeSH Major Topic]) AND "United States"[MeSH Terms] | Text Search: homicide AND ("United States" OR "US" OR "American" OR "USA") (Advanced), Search in: Projects Admin IC: All, Activity Code: R01, Fiscal Year: 2015 |

**^1^** Due to an issue with the search word ‘leukemia’ bringing up grants related solely to HIV, we used the boolean logic of ‘leukemia NOT HIV’ for the leukemia search.
