# Supplementary figures and images for: Do black lives matter in public health research and training?
Source: PLoS One. 2017 Oct 10;12(10):e0185957. doi: 10.1371/journal.pone.0185957 (PMC5634659; doi:10.1371/journal.pone.0185957)

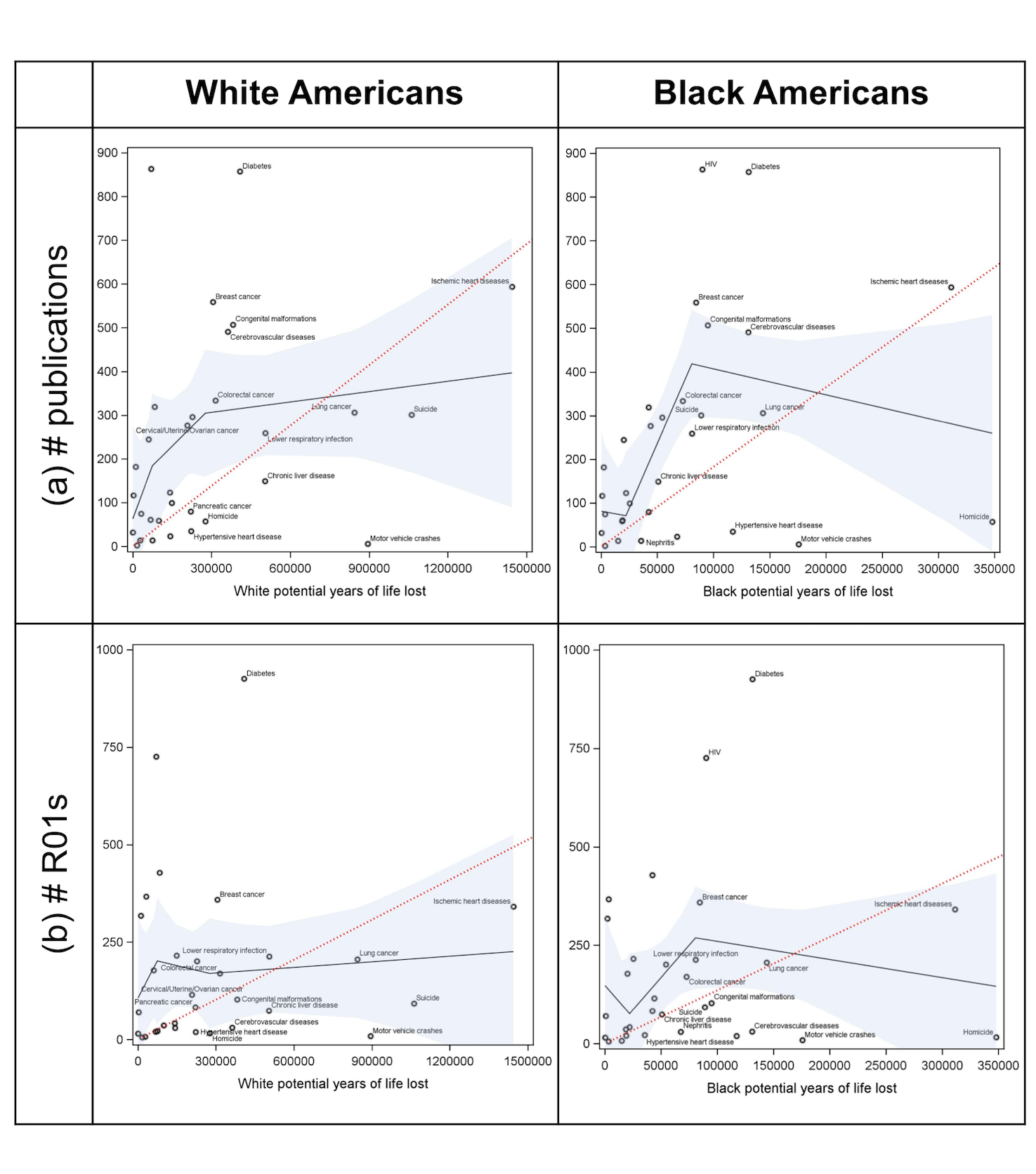

Supplement: S1 Fig — Solid black line shows predicted relationships from linear spline regression models with nodes at tertiles and 95% confidence limits in blue. Dotted red reference line shows hypothetical relationship if every 1% increase in PYLL corresponded with a 1% increase in (a) publications and (b) funded grants. Top 15 race-specific contributors to PYLL are labeled in each panel. (TIFF) [file pone.0185957.s003.tiff]
